# Dual graph convolutional networks integrating affective knowledge and position information for aspect sentiment triplet extraction

**DOI:** 10.3389/fnbot.2023.1193011

**Published:** 2023-08-14

**Authors:** Yanbo Li, Qing He, Damin Zhang

**Affiliations:** College of Big Data and Information Engineering, Guizhou University, Guiyang, China

**Keywords:** aspect-based sentiment analysis, aspect sentiment triplet extraction, affective knowledge, position-aware function, graph convolutional network (GCN)

## Abstract

Aspect Sentiment Triplet Extraction (ASTE) is a challenging task in natural language processing (NLP) that aims to extract triplets from comments. Each triplet comprises an aspect term, an opinion term, and the sentiment polarity of the aspect term. The neural network model developed for this task can enable robots to effectively identify and extract the most meaningful and relevant information from comment sentences, ultimately leading to better products and services for consumers. Most existing end-to-end models focus solely on learning the interactions between the three elements in a triplet and contextual words, ignoring the rich affective knowledge information contained in each word and paying insufficient attention to the relationships between multiple triplets in the same sentence. To address this gap, this study proposes a novel end-to-end model called the Dual Graph Convolutional Networks Integrating Affective Knowledge and Position Information (DGCNAP). This model jointly considers both the contextual features and the affective knowledge information by introducing the affective knowledge from SenticNet into the dependency graph construction of two parallel channels. In addition, a novel multi-target position-aware function is added to the graph convolutional network (GCN) to reduce the impact of noise information and capture the relationships between potential triplets in the same sentence by assigning greater positional weights to words that are in proximity to aspect or opinion terms. The experiment results on the ASTE-Data-V2 datasets demonstrate that our model outperforms other state-of-the-art models significantly, where the F1 scores on 14res, 14lap, 15res, and 16res are 70.72, 57.57, 61.19, and 69.58.

## 1. Introduction

In recent years, significant advancements in deep learning have been attributed to the development of more efficient algorithms, advancements in hardware capabilities, and the availability of extensive datasets. These progressions have paved the way for the emergence of diverse types of dynamic neural networks (DNN) tailored to address specific challenges across various domains. For instance, deep learning has been instrumental in surface defect recognition in the realm of computer vision (Shi et al., 2023), Artificial Intelligence (AI) systems based on deep learning algorithms can effectively detect and analyze arc faults in electrical systems (Tian et al., 2023) and recurrent neural networks (RNN) are designed to capture temporal dependencies and sequential patterns, thus making them well suited for tasks involving gesture recognition and classification. Moreover, the utilization of graph structures for learning purposes has demonstrated tremendous potential in various fields. For example, in the domain of blockchain technology, graph structure learning methods have been employed to enhance the analysis of transaction networks and identify the characteristics of the transaction (Wang et al., 2023). Additionally, improved graph structure learning methods (Liu et al., 2023) based on the foundational graph neural network (GNN) have been proposed in order to further enhance the capabilities of graph-based learning.

**Figure 1:**
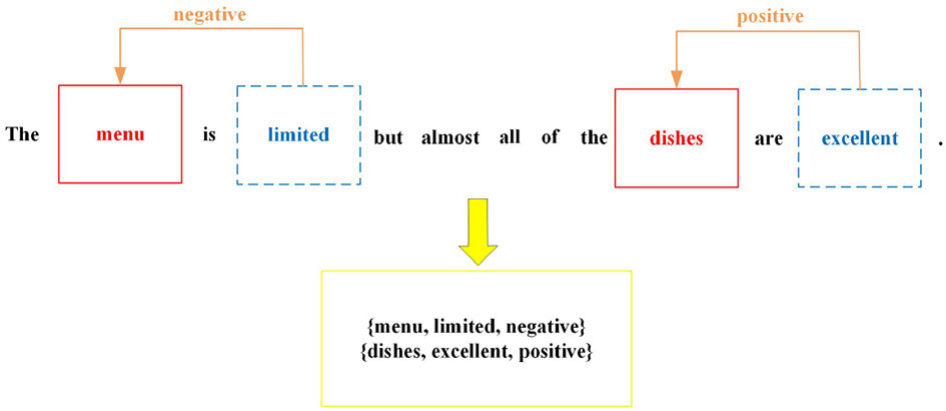
An example of ASTE. The aspect terms are highlighted in red. The terms in blue are opinion terms and the origin words that denote their sentiment polarity. All triplets are shown in the yellow box.

In the field of natural language processing (NLP), comments of consumers serve as a valuable resource for gathering information that can aid in enhancing the performance of robots and their associated products or services. With the proliferation of social media communities, the availability of consumer-generated content has expanded significantly, presenting an opportunity to leverage this data for insights and improvements. By employing methods designed for text information, robots can significantly enhance their ability to understand the intent and meaning behind a comment of consumer. These methods enable robots to extract the most valuable information from user input, leading to more accurate and meaningful interactions. Aspect Sentiment Triplet Extraction (ASTE) (Peng et al., 2020) is concerned with identifying the triplets from a given comment. Each triplet includes an aspect term, corresponding opinion term, and the sentiment polarity of this aspect term. For instance, in Figure 1, this comment from restaurant domain comprises two triplets: (*menu, limited, negative*) and (*dishes, excellent, positive*). Aspect sentiment triplet extraction plays a crucial role in enabling a more fine-grained understanding of text by capturing sentiments toward specific aspects or features. This capability facilitates context-aware analysis, supports decision-making processes, analyzes customer feedback, and aids in brand monitoring and reputation management.

Aspect Sentiment Triplet Extraction (ASTE) is a fine-grained task of Aspect-based Sentiment Analysis (ABSA) (Pontiki et al., 2014). ABSA aims to extract aspect terms and identify the corresponding sentiment polarity from a given sentence. It typically includes subtasks such as Aspect Terms Extraction (ATE) (Yin et al., 2016; Xin et al., 2018; Wu et al., 2020b), Opinion Terms Extraction (OTE) (Jebbara and Cimiano, 2017; Jordhy et al., 2019; Li et al., 2019), and Aspect-based Sentiment Classification (ASC) (Tang et al., 2016; Ma et al., 2017; He et al., 2018). ASTE is the combination of these subtasks and initially proposed in the study by Peng et al. (2020) with a two-stage pipeline approach. This method predicts all aspect terms, opinion terms, and sentiment polarities in the first stage. In the second stage, aspect terms are paired with their corresponding opinion terms to obtain triplets. However, this approach is susceptible to error propagation. To overcome this limitation, Xu et al. (2020) propose a position-aware tagging scheme and develop a union model that uses sequence labeling to extract triplets. This method is the first end-to-end model in the ASTE task. Similarly, Wu et al. (2020a) present a grid tagging scheme named GTS that uses a unified grid markup task to extract triplets in an end-to-end manner.

During sentiment analysis, it is observed that every word in a sentence possesses a unique emotional intensity. For instance, while words such as “likable” and “charming” both convey a positive sentiment polarity, their degrees of positivity differ. However, it has been noted that current networks relying on graph convolutional network tend to utilize solely syntactic dependencies for graph construction, thereby ignoring the commonsense knowledge information (Erik et al., 2009) associated with each word. Furthermore, such models typically overlook the relationships between multiple triplets present in the same sentence.

To overcome the aforementioned limitations of existing models, this study presents a novel approach that takes into account both affective knowledge information and the implicit relationship between different potential triplets in the same sentence. The proposed method employs a part-of-speech (POS) based approach to identify potential aspect terms and opinion terms within sentences, then formulates a fresh approach for generating an adjacency matrix, which fuses the affective score of each word from SenticNet (Ma et al., 2018) with the syntax dependency in two parallel modules, leading to the generation of a potential aspect terms enhanced adjacency matrix and a potential opinion terms enhanced adjacency matrix. These adjacency matrices are, then, input into a graph convolutional network (GCN) (Kipf and Welling, 2016) to extract features separately. GCN is a neural network architecture that has the ability to extract both contextual and syntactic representations from the adjacency matrix by aggregating the features of neighboring nodes. Additionally, this study utilizes a multi-target position-aware function in each GCN module, which assigns different weights to all words based on the position of potential aspect words or opinion words. This facilitates interaction between different potential triplets in a sentence and reduces interference from other words on triplet extraction. Finally, the hidden representations produced by the encoder layer, and two GCN modules are used *via* GTS for triplet extraction.

The main contributions of our study can be summarized as follows:

- We propose an innovative Dual Graph Convolutional Networks Integrating Affective Knowledge and Position Information (DGCNAP) for the ASTE task in an end-to-end manner.
- We conceive a novel method to introduce affective knowledge information into the adjacency matrix generated by sentences in the ASTE task.
- We design a multi-target position-aware function in the GCN layer to reduce interference and capture the associations between different potential triplets in the same sentence.
- Our experimental results on four benchmark datasets demonstrate the effectiveness of our model in the ASTE task.

## 2. Related work

Unlike traditional sentiment analysis that aims to identify the sentiment polarity of the whole document or sentence, ABSA aims to predict sentiment polarity of specific aspect terms. In recent research, most models use attention mechanisms. Wu et al. (2022) proposed a phrase dependency graph attention network to aggregate directed dependency edges and phrase information. Liang et al. (2022) adopted a graph convolutional network based on affective knowledge to leverage the affective dependencies of the sentence; thus, both the dependencies of contextual words and aspect words and the affective information between opinion words and the aspect are considered.

To establish a comprehensive solution for ABSA, ASTE aims to complete multiple subtasks of ABSA simultaneously. In the ASTE task, existing methods can be divided into two types: pipeline methods and end-to-end methods. Peng et al. (2020) are the first to propose a complete solution for the ASTE task, employing a two-stage pipeline approach. However, models constructed using this pipeline approach are rather simple and are easily affected by error propagation. To avoid this problem, end-to-end models have been proposed and can be summarized as follows. Xu et al. (2020) first developed an end-to-end method named position-aware tagging scheme. Similarly, Wu et al. (2020a) proposed grid tagging scheme to extract triplets simultaneously. Considering ASTE is the combination of all basic tasks of ABSA, Chen et al. (2022) proposed an end-to-end approach which decomposes ASTE into three subtasks, namely, target tagging, opinion tagging, and sentiment tagging. Chen et al. (2021) proposed a novel method which transforms ASTE task into a multi-turn machine reading comprehension task and propose a bidirectional MRC framework to address this challenge. Another end-to-end method (Dai et al., 2022) proposed a sentiment-dependence detector based on a dual-table structure that starts from two directions, aspect-to-opinion and opinion-to-aspect, to generate two sentiment-dependence tables dominated by two types of information. Shi et al. (2022) proposed an interactive attention mechanism to jointly consider both the contextual features and the syntactic dependencies in an iterative interaction manner. Previous tag-based joint extraction methods have been observed to struggle with effectively handling one-to-many and many-to-one relationships between aspect terms and opinion terms within sentences. This limitation has motivated researchers to explore alternative approaches, such as those that operate at the span level rather than relying on tagging schemes. A tagging-free approach (Mukherjee et al., 2021) is proposed to capture the span-level semantics while predicting the sentiment between an aspect-opinion pair. Li et al. (2022) proposed a span-sharing joint extraction framework to extract aspect terms and their corresponding opinion terms simultaneously in the last step, thereby avoiding error propagation. Hu et al. (2023) used a span GCN for syntactic constituency parsing tree and a relational GCN (R-GCN) for commonsense knowledge graph to build an end-to-end model for the ASTE task. Moreover, a double-embedding mechanism-character-level and word-vector embeddings are introduced for the first time. Zhang et al. (2022) propose a dual convolutional neural network with a span-based tagging scheme to extract multiple entities directly under the supervision of span boundary detection.

## 3. Approach

Existing models have achieved good performance on the ASTE task. However, a significant number of these methods disregard the abundant affective knowledge present in individual words of a sentence, as well as the interdependence of various triplets. To address this limitation, we introduce affective knowledge information in our framework while constructing the dependency graph. Additionally, we utilize a multi-target position-aware function to capture the interdependence of multiple triplets in the same sentence, and it can also mitigate the adverse effects of noisy words.

This section commences with a definition of the ASTE task followed by an elaborate elucidation of our proposed methodology, Dual Graph Convolutional Networks Integrating Affective Knowledge and Position Information (DGCNAP), for the ASTE task.

### 3.1. Definition of ASTE

Given an n-word sentence $S=\left\{w_{1},w_{2},...,w_{n}\right\}$, the ASTE task aims at identifying all sentiment triplet sets T={at,ot,s}, where “at” denotes the aspect term, “ot” denotes the opinion term, “s” denotes the sentiment of the aspect term in this set, and *s*∈{*positive, negative, neutral*}.

### 3.2. The DGCNAP framework

**Figure 2:**
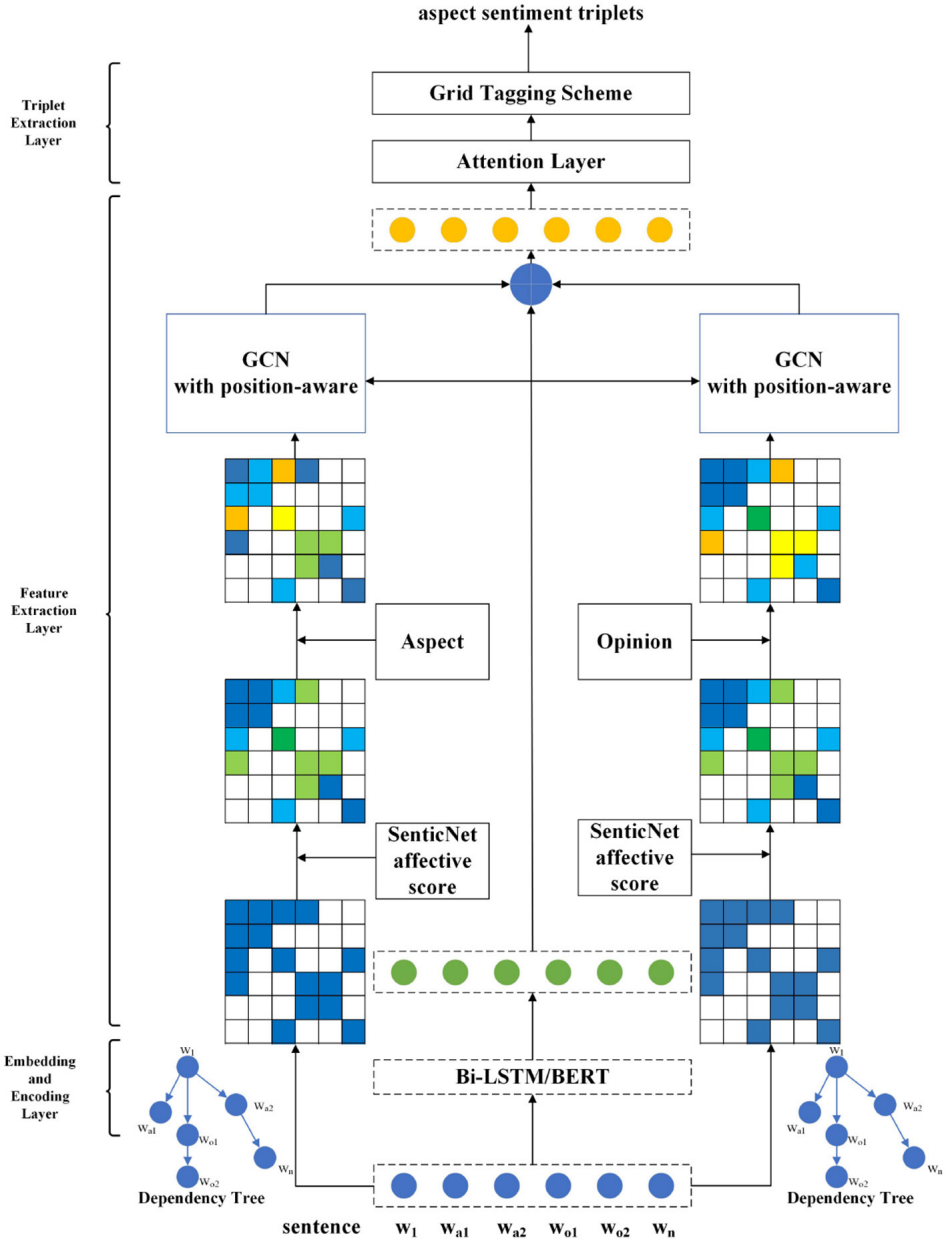
Architecture of DGCNAP.

The overall architecture of DGCNAP model is shown in Figure 2. The model takes two parallel channels to joint potential aspect term and potential opinion term enhanced features extraction, leveraging affective knowledge, graph convolutional network, and multi-target position-aware function to improve accuracy and capture the complex relationships between aspect and opinion terms in sentences.

### 3.3. Embedding and encoding layers

In this study, we employ two types of encoders to learn hidden representations: the first is the Bi-directional Long Short-Term Memory (Bi-LSTM) (Hochreiter and Schmidhuber, 1997) network and the second is the pre-trained language model BERT (Devlin et al., 2019).

For the Bi-LSTM-based encoder, we utilize double embedding to obtain the initial word representation and capture the contextual meaning of words in a specific domain. The specific-domain embedding was pre-trained based on the skip-gram model, where each word is represented as a bag of character n-grams. A vector representation is associated with each character n-gram; words are represented as the sum of these representations. We concatenate the 300-dimension general-domain embedding $E_{w}\in {\mathbb{R}}^{n\times d_{w}}$ and the 100-dimension specific-domain embedding $E_{s}\in {\mathbb{R}}^{n\times d_{s}}$ to form the final word representation $E\in {\mathbb{R}}^{n\times \left(d_{w}+d_{s}\right)}$, where *d*_*w*_ and *d*_*s*_ denote the dimensions of word embedding. After that, we input the embedding matrix into a Bi-LSTM to obtain the hidden contextual representations $H^{c}=\left\{h_{1},h_{2},...,h_{n}\right\}\in {\mathbb{R}}^{n\times d_{I}}$ of the input sentence, where *d*_*I*_ denotes the hidden state dimension of Bi-LSTM:


(1) $$\begin{array}{l} H^{c}=Bi-LSTM\left(E\right) \end{array}$$

For the BERT-based encoder, we first add the [CLS] token at the beginning of the sentence and the [SEP] token at the end. Next, we feed the sequence into BERT for context encoding by converting it into a vector that sums its token embedding, segment embedding, and position embedding. Finally, we input the vector *v* into the transformer encoder (Vaswani et al., 2017), to obtain the hidden contextual representation $H^{c}=\left\{h_{1},h_{2},...,h_{n}\right\}\in {\mathbb{R}}^{n\times d_{I}}$:


(2) $$\begin{array}{l} H^{c}=BERT\left(v\right) \end{array}$$

### 3.4. Generate enhanced graph

**Figure 3:**
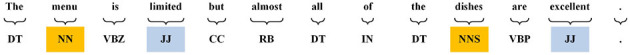
An example of part-of-speech tagging.

Part-of-speech (POS) is a linguistic concept that categorizes words based on their grammatical roles and syntactic functions within a sentence. Each word in a sentence is assigned a specific part-of-speech tag, which provides information about its linguistic characteristics and relationships with other words. As shown in Figure 3, the aspect terms “menu” and “dishes” are both annotated as nouns, and the opinion terms “limited” and “excellent” are both annotated as adjectives. In the proposed approach, nouns are considered as potential aspect terms, while adjectives are identified as potential opinion terms.

Dependency graph is a useful way to represent the grammatical relationships between words in a sentence. We use the dependency tree of each input sentence to construct a unidirectional dependency graph with self-loop. *D*∈ℝ^*n*×*n*^ denotes the adjacency matrix obtained from the graph:


(3) $$D_{i,j}=\{\begin{array}{l} 1\;{\text{if w}}_{\text{i}}{\text{ and w}}_{\text{j}}\text{ contains dependency}\\ 0\text{ otherwise} \end{array}$$

Because the parent node is also affected by the child node, *D*_*j, i*_ = *D*_*i, j*_.

To incorporate affective knowledge into the construction of the dependency graph, we take the absolute value of the SenticNet affective score and use it as a weight for the corresponding edge in the adjacency matrix. By doing so, we can assign more weight to words with stronger sentiment intensity when computing the graph convolution operation, and our model can learn meaningful information from words containing emotionally intense, thereby contributing to increased accuracy in predicting sentiment polarity corresponding to aspect terms:


(4) $$\begin{array}{l} S_{i,j}=|SenticNet\text{(}w_{i}\text{)}|+|SenticNet\text{(}w_{j}\text{)}| \end{array}$$

where *SenticNet*(*w*_*i*_)∈[−1, 1] denotes the SenticNet affective score of word *w*_*i*_. When *SenticNet*(*w*_*i*_) approaches -1, the word conveys a strong negative sentiment. Conversely, as *SenticNet*(*w*_*i*_) approaches 1, the word expresses a strong positive sentiment. In cases where *SenticNet*(*w*_*i*_) is equal to 0, the word *w*_*i*_ is considered neutral or is not included in the SenticNet database. We exploit SenticNet 6, which contains 200,000 concepts. Some examples of SenticNet are shown in Table 1.

**Table 1:** Examples of SenticNet.

**Word**	**SenticNet(word)**
Distrustful	-0.93
Undesirable	-0.35
Likable	0.301
Charming	0.885

To enhance the sentiment dependencies that exist between potential aspect words and contextual words, as well as between potential opinion words and contextual words, we incorporate potential aspect word weights and potential opinion word weights as the target score into the generation of the adjacency matrix:


(5) $$T_{i,j}^{a}=\{\begin{array}{l} 1\;{\text{if w}}_{\text{i}}{\text{ or w}}_{\text{j}}\text{ is a potential aspect word}\\ 0\text{ otherwise} \end{array}$$

(6) $$T_{i,j}^{o}=\{\begin{array}{l} 1\;{\text{if w}}_{\text{i}}{\text{ or w}}_{\text{j}}\text{ is a potential opinion word}\\ 0\text{ otherwise} \end{array}$$

To learn the syntactic information features enhanced by aspect words and opinion words, respectively, we employ two parallel channels. The first channel generates an adjacency matrix that has been augmented by both aspect words and SenticNet affective score, whereas the second channel generates an adjacency matrix that has been enhanced by both opinion words and SenticNet affective score. To effectively integrate the SenticNet affective score with the aspect word weight or opinion word weight, we use the following formula to generate the final enhanced adjacency matrix $A_{i,j}^{a}$ and $A_{i,j}^{o}$:


(7) $$\begin{array}{l} W_{i,j}^{a}=D_{i,j}+S_{i,j}+T_{i,j}^{a} \end{array}$$

(8) $$\begin{array}{l} A_{i,j}^{a}=\frac{1-e^{-2\times W_{i,j}^{a}}}{1+e^{-2\times W_{i,j}^{a}}}+0.23841 \end{array}$$

(9) $$\begin{array}{l} W_{i,j}^{o}=D_{i,j}+S_{i,j}+T_{i,j}^{o} \end{array}$$

(10) $$\begin{array}{l} A_{i,j}^{o}=\frac{1-e^{-2\times W_{i,j}^{o}}}{1+e^{-2\times W_{i,j}^{o}}}+0.23841 \end{array}$$

When encountering a word that is neither a potential aspect word nor a potential opinion word, and its corresponding SenticNet affective score is 0, the utilization of the bias value of 0.23841 results in an output of 1, with consideration to the precision of five decimal places.

### 3.5. Feature extraction layer

A two-layer GCN is utilized for contextual feature extraction in each channel. The syntactic dependencies for the potential aspect words or opinion words are captured by feeding the enhanced adjacency matrix *A*^*a*^∈ℝ^*n*×*n*^ and the hidden contextual representations $H^{c}\in {\mathbb{R}}^{n\times d_{I}}$ into the GCN module in the left channel. Additionally, the enhanced adjacency matrix *A*^*o*^∈ℝ^*n*×*n*^ and the hidden contextual representations $H^{c}\in {\mathbb{R}}^{n\times d_{I}}$ are input into the GCN module of another channel. Inspired by (Zhang et al., 2019), prior to this convolution, we utilize the hidden contextual representations $H^{c}\in {\mathbb{R}}^{n\times d_{I}}$ as input into the multi-target position-aware function ${ℱ}^{a}$ and ${ℱ}^{o}$ to augment the importance of context words close to the potential aspect words or opinion words in two separate channels. Considering that there may be multiple potential aspect terms and opinion terms in one sentence, the function is as follows:


(11) $$\begin{array}{l} q_{i}^{t}=\{\begin{array}{ll} 1-\frac{\tau +1-i}{n} & \text{1 }⩽\text{ i}<\tau +\text{1}\\ 0 & \tau +\text{1 }⩽\text{ i }⩽\;\tau +m\\ 1-\frac{i-\tau +1-m}{n}\; & \tau +m<\text{i }⩽\text{ n} \end{array} \end{array}$$

(12) $${ℱ}^{a}(h_{i}^{l})=\{\begin{array}{ll} \frac{q_{i}^{1}+q_{i}^{2}+...+q_{i}^{t}}{t}h_{i}^{l} & {\text{if w}}_{\text{i}}\text{ is not a potential aspect word}\\ 0 & \text{otherwise} \end{array}$$

(13) $${ℱ}^{o}(h_{i}^{l})=\{\begin{array}{ll} \frac{q_{i}^{1}+q_{i}^{2}+...+q_{i}^{t}}{t}h_{i}^{l} & {\text{if w}}_{\text{i}}\text{ is not a potential opinion word}\\ 0 & \text{otherwise} \end{array}$$

where $q_{i}^{t}\in \mathbb{R}$ is the position weight to i-th token for the t-th potential aspect term or opinion term in the sentence in two parallel channels, respectively. This function enables the model to effectively avoid noise generated during dependency parsing, resulting in improved performance and more accurate capture of the relevant syntactic dependencies.

The process of GCN is as follows:


(14) $$\begin{array}{l} h_{i}^{l}=ReLu\left(Ag_{i}^{l-1}W^{l}+b^{l}\right) \end{array}$$

(15) $$g_{i}^{l-1}=ℱ(h_{i}^{l-1})$$

where $h_{i}^{l}$ denotes the output of the l-th GCN layer. The output of potential aspect term-enhanced GCN layer is $H^{a}\in {\mathbb{R}}^{n\times d_{I}}$, and the output of potential opinion term-enhanced GCN layer is $H^{o}\in {\mathbb{R}}^{n\times d_{I}}$. After that, the final output of Features Extraction Layer *H* can be computed as follow:


(16) $$\begin{array}{l} H=H^{c}+H^{a}+H^{o}=\left\{\tilde{h_{1}},\tilde{h_{2}},...,\tilde{h_{n}}\right\} \end{array}$$

### 3.6. Triplet extraction layer

**Figure 4:**
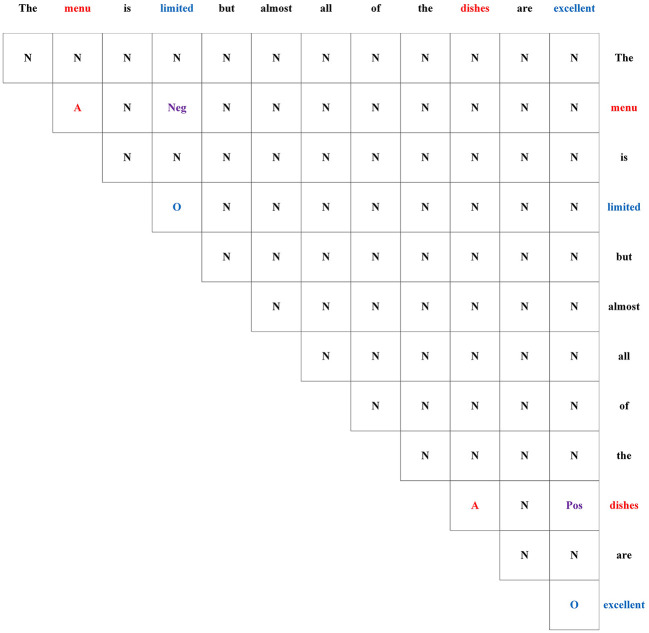
A tagging example with GTS.

In previous research (Wu et al., 2020a), GTS has been demonstrated to be a highly effective module for extracting triplets from the ASTE task. Therefore, in this study, we have adopted GTS as the decoding algorithm in our proposed model. The output of the Features Extraction Layer is passed through a self-attention layer to extract high-level features. The resulting output is, then, fed into the GTS module. In the GTS module, the relation of two words of the sentence is tagged by set {*A, O, Pos, Neu, Neg, N*}. Specifically, the symbols “A” and “O” indicate that the two terms belong to the same triplet, and that they are an aspect term and an opinion term, respectively. The tags “Pos,” “Neu,” and “Neg” denote the sentiment polarity of the triplet. The symbol “N” represents that there is no association between the two words.An example of the GTS tagging scheme is shown in Figure 4. The following inference strategy is used to predict probability distribution $p_{i}j^{t}$ of word pair (*w*_*i*_, *w*_*j*_) as follows:


(17) $$\begin{array}{l} p_{i}^{t-1}=maxpooling\left(p_{i,:}^{t-1}\right) \end{array}$$

(18) $$\begin{array}{l} p_{j}^{t-1}=maxpooling\left(p_{j,:}^{t-1}\right) \end{array}$$

(19) $$\begin{array}{l} q_{ij}^{t-1}=\left[z_{ij}^{t-1};p_{i}^{t-1};p_{j}^{t-1};p_{ij}^{t-1}\right] \end{array}$$

(20) $$\begin{array}{l} z_{ij}^{t}=W_{q}q_{ij}^{t-1}+b_{q} \end{array}$$

(21) $$\begin{array}{l} p_{ij}^{t}=softmax\left(W_{s}z_{ij}^{t}+b_{s}\right) \end{array}$$

where *W*_*q*_, *W*_*s*_, *b*_*q*_, and *b*_*s*_ are learnable parameters, $p_{i}^{t-1}$ represents all predicted probability between the word *w*_*i*_ and other words, t denotes the t-th inference, and [.;.] represents the vector concatenation operation. The first three equations are used to observe the probability distribution characteristics of each word pair itself and between word pairs. The initial predicted probability $p_{ij}^{0}$ and representation $z_{ij}^{0}$ of word pair (*w*_*i*_, *w*_*j*_) are set as follows:


(22) $$\begin{array}{l} p_{ij}^{0}=softmax\left(W_{s}r_{ij}^{t}+b_{s}\right) \end{array}$$

(23) $$\begin{array}{l} z_{ij}^{0}=r_{ij} \end{array}$$

where $r_{ij}=\left[\tilde{h_{i}};\tilde{h_{j}}\right]$. Finally, the prediction of the last round is used to extract triplets. The decoding algorithm first predicts aspect terms and opinion terms based on the tags on the main diagonal. It, then, determines whether there are any terms among them that can form a pair. Finally, the most predicted sentiment tag is selected as the sentiment polarity of the pair, and the resulting pair and sentiment polarity are combined to form a triplet.

### 3.7. Loss function

We use the loss function which defined as cross entropy loss between the real label and the predicted label of all word pairs, and the training goal is to minimize it as follows:


(24) $$ℒ=-\sum_{i=1}^{n}\sum_{j=1}^{n}\sum_{k\in c}I(y_{ij}=k)log(P_{i,j|k}^{L})$$

## 4. Experiments

### 4.1. Datasets

In this study, we have conducted experiments on three public benchmark datasets from the restaurant domain and a public benchmark dataset from laptop domain named ASTE-Data-V2 mentioned in the study by Xu et al. (2020), all of which have been sourced from the SemEval Challenges and contain 5,989 different comments. Additionally, we have also carried out experiments on the ASTE-Data-V1 datasets mentioned in the study by Wu et al. (2020a) and report the results of these experiments. The details of these datasets are shown in Tables 2, 3.

**Table 2:** Statistics of the ASTE-Data-V1 datasets.

**Datasets**	**14res**			**14lap**			**15res**			**16res**		
	**Train**	**Dev**	**Test**	**Train**	**Dev**	**Test**	**Train**	**Dev**	**Test**	**Train**	**Dev**	**Test**
Sentences	1,259	315	493	899	225	332	603	151	325	863	216	328
Triplets	2,356	580	1,008	1,452	383	547	1,038	239	493	1,421	348	525

**Table 3:** Statistics of the ASTE-data-V2 datasets.

**Datasets**	**14res**			**14lap**			**15res**			**16res**		
	**Train**	**Dev**	**Test**	**Train**	**Dev**	**Test**	**Train**	**Dev**	**Test**	**Train**	**Dev**	**Test**
Sentences	1,266	310	492	906	219	328	605	148	322	857	210	326
Triplets	2,338	577	994	1,460	346	543	1,013	249	485	1,394	339	514

### 4.2. Evaluation metrics

To ensure the accuracy of the model's performance, Precision (P), Recall (R), and F1 Score (F1) are selected as the evaluation metrics, consistent with prior research in this field:


(25) $$\begin{array}{l} P=\frac{TP}{TP+FP} \end{array}$$

(26) $$\begin{array}{l} R=\frac{TP}{TP+FN} \end{array}$$

(27) $$\begin{array}{l} F1=\frac{2\times P\times R}{P+R} \end{array}$$

where “TP” denotes the number of the positive cases correctly predicted, and “TN” represents the number of negative cases correctly predicted. By contrary, “FP” represents the number of negative cases incorrectly predicted, and “FN” refers to the number of positive cases incorrectly predicted. Notably, the evaluation of extracted triplets is contingent upon the correct prediction of these three components, and any incorrectness in any of these components will render the triplet as incorrect.

### 4.3. Experiments settings

For the purpose of comparison with previous research, for the Bi-LSTM contextual encoder, following the design of GTS, we use a 300-dimension general-domain embedding from GloVe (Pennington et al., 2014) with 840 billion tokens and a 100-dimension specific-domain embedding from fastText (Bojanowski et al., 2017) to initialize the word embeddings. The hidden state size of the Bi-LSTM is 300, and the dimension is set to 50. The dropout rate of embedding is set to 0.3. For the BERT-based encoder, the bert-base-uncased is used as encoder, and it contains 12 attention mechanism heads, 12 hidden layers, and 768 hidden units. For these two types of encoders, we set Adam optimizer (Kingma and Ba, 2014) to optimize networks with an initial learning rate of 0.001 for the Bi-LSTM contextual encoder and 5e-5 for the BERT-based encoder. The hidden state size of the GCN is set to 300, and the depth of GCN layer is 2. The batch size is set to 32. We conducted 5 independent runs with randomized initialization and reported the experimental results as the average of these five runs.

### 4.4. Baselines

To evaluate the effectiveness of DGCNAP in the ASTE task, we present other state-of-the-art models in this task for comparison. These models can be categorized into end-to-end models and pipeline models.

**Pipeline models**

- **CMLA+ (Peng et al.**, **2020****)** is a two-stage model based on CMLA (Wang et al., 2017). In the first stage, it extracts aspect terms, opinion terms, and sentiment polarities through a multi-layer attention network. In the second stage, it generates possible triplets based on the output of the first stage, then utilizes a binary classifier to filter out invalid triplets.
- **RINANTE+ (Peng et al.**, **2020****)** is a two-stage model based on RINANTE (Dai and Song, 2019). The only difference between RINANTE+ and CMLA+ is that RINANTE+ extract aspect terms, opinion terms, and triplets through dependency parsing.
- **Li-Unified-R (Peng et al.**, **2020****)** is a two-stage framework based on Li-Unified (Li et al., 2019). In the first stage, it uses a customized multi-layer LSTM network to extract targets, opinions, and sentiments. The second stage is similar to CMLA+.
- **Peng + PD (Peng et al.**, **2020****)** is a pipeline model. It first predicts all possible triplets, then utilize a MLP classifier to judge the rationality of each triplet.
- **Peng + LOG (Wu et al.**, **2020a****)** is a pipeline model. The author add a model proposed in the study by (Fan et al., 2019), after the model proposed in the study by (Peng et al., 2020).
- **IMN-IOG (Wu et al.**, **2020a****)** is the combination of the IMM (He et al., 2019) and IOG (Fan et al., 2019) to generate triplets.

**End-to-end models**

- **OTE-MTL (Zhang et al.**, **2020****)** is a model that splits the ASTE task into multiple subtasks, then generate triplets through a bi-affine scorer.
- **JET (Xu et al.**, **2020****)** is a unified framework based on the position-aware tagging scheme to generate triplets through an LSTM layer and a CRF layer.
- **GTS (Wu et al.**, **2020a****)** is a model that generates triplets by a unified tagging scheme, and the authors design an effective inference strategy to exploit mutual indication between different opinion factors for more accurate extractions.
- **PASTE (Mukherjee et al.**, **2021****)** is a tagging-free solution built on an encoder–decoder architecture to produce all triplets.
- **UniASTE (Chen et al.**, **2022****)** is a multi-task learning framework which decompose ASTE into three subtasks.
- **GCN-EGTS (Hu et al.**, **2023****)** is an end-to-end model which is an enhanced Grid Tagging Scheme (GTS) for ASTE, leveraging syntactic constituency parsing tree and a commonsense knowledge graph based on GCNs.
- **DGEIAN (Shi et al.**, **2022****)** is a framework with an interactive attention mechanism. In addition, the authors add different part-of-speech categories in embedding layer.

### 4.5. Experimental results

The results of our proposed model in the ASTE task are presented in Tables 4, 5. From the results, it is clear that DGCNAP significantly outperforms all other models in terms of F1 score on all datasets. The observations in Table 4 represent that our DGCNAP also performs better than other baseline models on ASTE-Data-V1 datasets. Our method outperforms DGEIAN on the four datasets and acquires 2.36, 1.12, 0.54, and 2.05 improvements in the F1, respectively. Additionally, we observe that the end-to-end model achieves better performance than the pipeline model. For the Bi-LSTM-based encoder, as shown in Table 5, when compared with the best pipeline model, Peng + PD, DGCNAP achieves F1 scores that are more than 10 percentage points higher in three out of the four datasets. On the other hand, in comparison with the model, our proposed model outperforms it by 2.83, 3.7, 1.55, and 3.62 F1 points on the respective datasets. For the BERT-based encoder, DGCNAP also performs well. From the Table 4, it can be observed that the DGCNAP outperforms by 0.06, 4.16, 0.82, and 3.19 F1 points on four datasets when compared with GTS. Our method outperforms the best BERT-based baseline model UniASTE by 1.63, 1.06, 2.14, and 2.36 F1 points, as shown in Table 5. The comparisons presented above demonstrate that our model effectively leverages the affective knowledge information of individual words, leading to improved model's performance in handling sentences with multiple triplets.

**Table 4:** Statistics of the ASTE-Data-V1 datasets.

**Encoder**	**Methods**	**14res**			**14lap**			**15res**			**16res**		
		**P**	**R**	**F1**	**P**	**R**	**F1**	**P**	**R**	**F1**	**P**	**R**	**F1**
Bi-LSTM	Peng + LOG^†^	58.89	60.41	59.64	48.62	45.52	47.02	51.70	46.04	48.71	59.25	58.09	58.67
	IMN + IOG^†^	59.57	63.88	61.65	49.21	46.23	47.68	55.24	52.33	53.75	-	-	-
	GTS-CNN^†^	70.79	61.70	65.95	55.93	**47.52**	51.38	60.09	**53.57**	56.64	62.63	**66.98**	64.73
	GTS-BiLSTM^†^	67.28	61.91	64.49	59.42	45.13	51.30	63.26	50.71	56.29	66.07	65.05	65.56
	GCN-EGTS(CNN)	68.74	62.07	65.72	55.94	45.25	49.89	61.54	51.29	55.97	63.73	63.86	63.77
	DGEIAN	71.03	62.63	66.55	60.74	45.56	51.72	**64.87**	52.75	57.11	69.07	65,64	67,30
	**DGCNAP**	**74.51**	**64.10**	**68.91**	**62.02**	46.09	**52.84**	64.82	51.92	**57.65**	**73.97**	65.29	**69.35**
BERT	GCN-EGTS_*BERT*_	70.14	68.07	69.20	54.54	52.27	53.64	59.23	**58.15**	58.84	66.89	65.86	66.28
	GTS_*BERT*_	70.92	**69.49**	70.20	57.52	51.92	54.58	59.29	58.07	58.67	68.58	66.60	67.58
	**DGCNAP**_***BERT***_	**71.83**	68.77	**70.26**	**63.91**	**54.34**	**58.74**	**62.03**	57.18	**59.49**	**69.39**	**72.20**	**70.77**

The best results are in bold. The results with “†” are retrieved from the study by Shi et al. (2022), others are retrieved from the original studies.

**Table 5:** Statistics of the ASTE-Data-V2 datasets.

**Encoder**	**Methods**	**14res**			**14lap**			**15res**			**16res**		
		**P**	**R**	**F1**	**P**	**R**	**F1**	**P**	**R**	**F1**	**P**	**R**	**F1**
Bi-LSTM	CMLA ^†^	39.18	47.13	42.97	30.39	36.92	33.16	34.56	39.84	37.01	41.34	42.10	41.72
	RINANTE +^†^	31.42	39.38	34.95	21.72	18.66	20.07	29.88	30.06	29.97	25.68	22.30	23.87
	Li-unified-R^†^	41.04	**67.35**	51.00	40.56	44.28	42.34	44.72	51.39	47.82	37.33	54.51	44.31
	Peng + PD^†^	43.24	63.66	51.46	37.38	**50.38**	42.87	48.07	**57.51**	52.32	46.96	64.24	54.21
	OTE-MTL^†^	63.00	55.10	58.70	49.20	40.50	45.10	57.90	42.70	48.90	60.30	53.40	56.50
	JET(M=6)^†^	61.50	55.13	58.14	53.03	33.89	41.35	64.37	44.33	52.50	70.94	57.00	63.21
	PASTE-AF^†^	62.40	61.80	62.10	53.70	48.60	51.00	54.80	53.40	54.10	62.20	62.80	62.50
	PASTE-OF^†^	63.40	61.90	62.60	59.70	48.10	50.00	54.80	52.60	53.70	62.30	63.60	62.90
	UniASTE	70.23	56.82	62.73	55.64	40.91	47.11	63.09	48.37	54.73	66.34	59.26	62.58
	DGEIAN	71.68	61.62	66.26	60.15	43.44	51.14	61.84	50.99	55.89	69.40	60.15	64.37
	**DGCNAP**	**74.43**	64.49	**69.09**	**64.32**	47.84	**54.84**	**66.73**	50.43	**57.44**	**72.37**	**64.13**	**67.99**
BERT	JET(M = 6)_*BERT*_	70.56	55.94	62.40	55.39	47.33	51.04	64.45	51.96	57.53	**70.42**	58.37	63.83
	UniASTE_*BERT*_	72.14	66.30	69.09	**62.24**	51.77	56.51	**64.83**	54.31	59.05	69.06	65.53	67.22
	**DGCNAP**_***BERT***_	**72.90**	**68.69**	**70.72**	62.02	**53.79**	**57.57**	62.23	**60.21**	**61.19**	69.75	**69.44**	**69.58**

The best results are in bold. The results with “^†^” are retrieved from the study by (Shi et al., 2022), others are retrieved from the original studies.

### 4.6. Ablation study

To investigate the effectiveness of the various components in our proposed model, we conducted a series of ablation experiments on the ASTE-data-V2 datasets using the Bi-LSTM encoder. The results of the ablation experiments are presented in Table 6. “w/o SN” refers to the adjacency matrix that is generated only by sentence dependency syntax, without adding SenticNet affective score to the adjacency matrix, and “w/o PA” indicates the model without the multi-target position-aware function in the GCN layer. “w/o AE” and “w/o OE” correspond to the models without the aspect words-enhanced GCN channel and the opinion words-enhanced GCN channel, respectively.

**Table 6:** Results of ablation study under the metric of F1.

**Model**	**14res**	**14lap**	**15res**	**16res**
DGCNAP	69.09	54.84	57.44	67.99
w/o SN	68.17	52.07	56.98	66.76
w/o PA	64.59	49.52	56.03	64.73
w/o AE	68.59	53.84	57.02	66.89
w/o OE	68.35	53.49	56.57	66.43

Based on the results of the ablation experiments presented in Table 6, we can draw the following conclusion. First, the SenticNet affective score is a crucial component in enhancing the representation of the dependency graph. The utilization of only the adjacency matrix generated from the dependency syntax tree, without incorporating the SenticNet affective score for enhancement, leads to a reduction in the model's ability to predict sentiment polarity. Second, the multi-target position-aware function is another critical module in our proposed model. The removal of this function leads to a significant decrease in the F1 score, the F1 score drops the most to 5.32 on the 14lap dataset, further highlighting the importance of this function in our model. Finally, the ablation experiments reveal that both the aspect terms-enhanced features and the opinion terms-enhanced features are important for model learning. The removal of either of these two channels leads to an average decrease by 0.76 and 1.13 F1 points, emphasizing their contribution to the overall performance of the DGCNAP model.

### 4.7. Impact of SenticNet effective score

**Figure 5:**
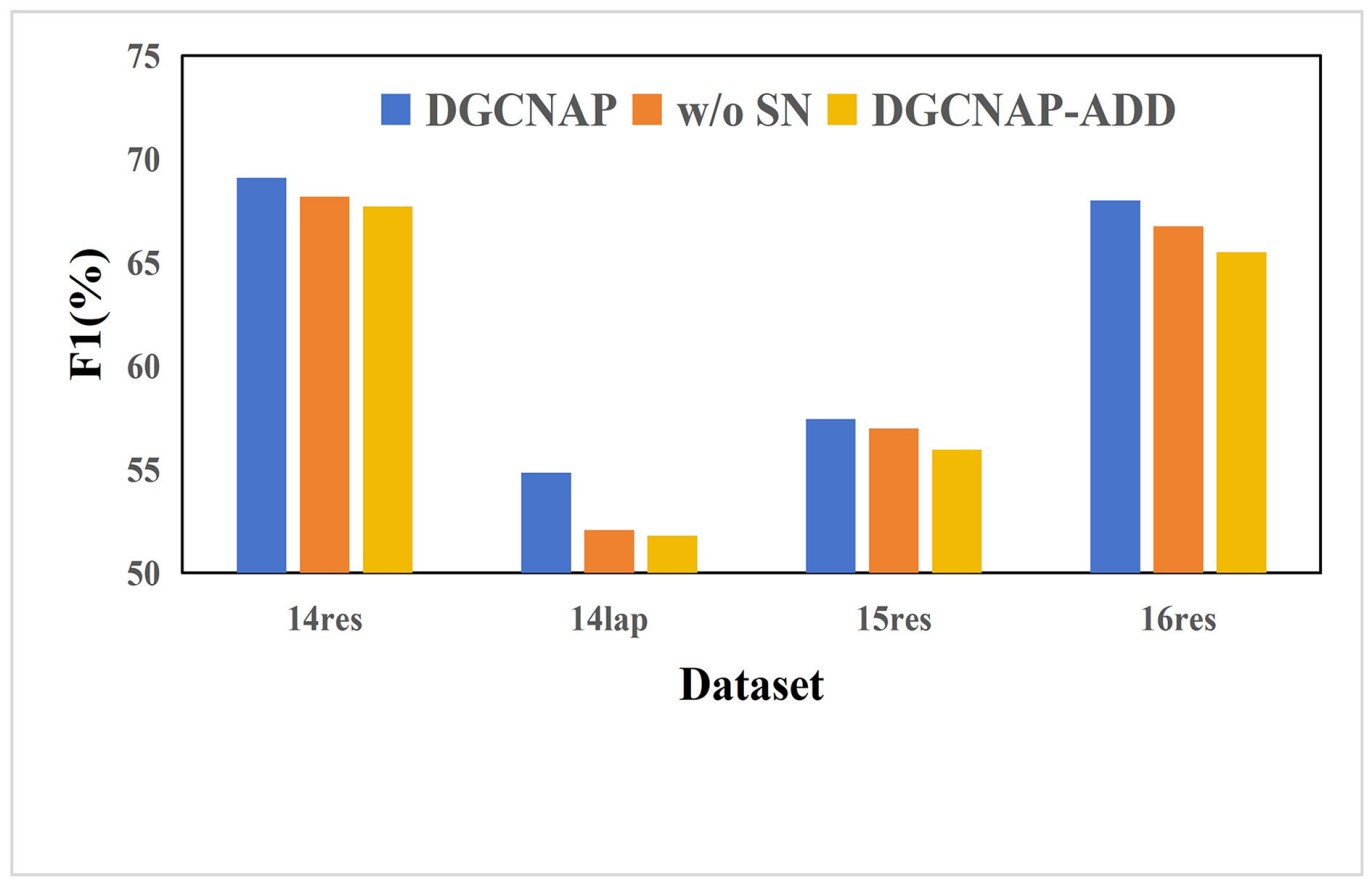
F1 scores for different use methods of SenticNet effective score on ASTE-data-V2 datasets.

To investigate the impact of incorporating SenticNet affective score, a series of experiments are conducted on all four ASTE-data-V2 datasets using Bi-LSTM encoder. Specifically, the aim is to explore the impact of using different strategies for incorporating SenticNet effective score. Furthermore, “DGCNAP-ADD” denotes that we generate the final weight of the enhanced graph which is generated by adding the weight of the adjacency matrix to the target score and the absolute value of the SenticNet affective score. The results of the experiments are presented in Table 7, and the corresponding F1 scores are plotted in Figure 5. The experimental results reveal that direct addition of the three values without proper processing during the generation of the final dependency matrix lead to overemphasis of the target words and words with strong emotions. Consequently, the model disregarded the impact of syntactic dependencies and semantic information, leading to undesirable side effects, and resulting in lower performance than the result before adding target weight and SenticNet effective score. Therefore, it is concluded that the incorporation of SenticNet affective score should be carried out with caution as inappropriate usage could have a negative impact on the performance of the model.

**Table 7:** Results of the different usage of SenticNet effective score under the metric of F1.

**Model**	**14res**	**14lap**	**15res**	**16res**
DGCNAP	69.09	54.84	57.44	67.99
w/o SN	68.17	52.07	56.98	66.76
DGCNAP-ADD	67.70	51.80	55.96	65.51

### 4.8. Impact of position-aware function

**Figure 6:**
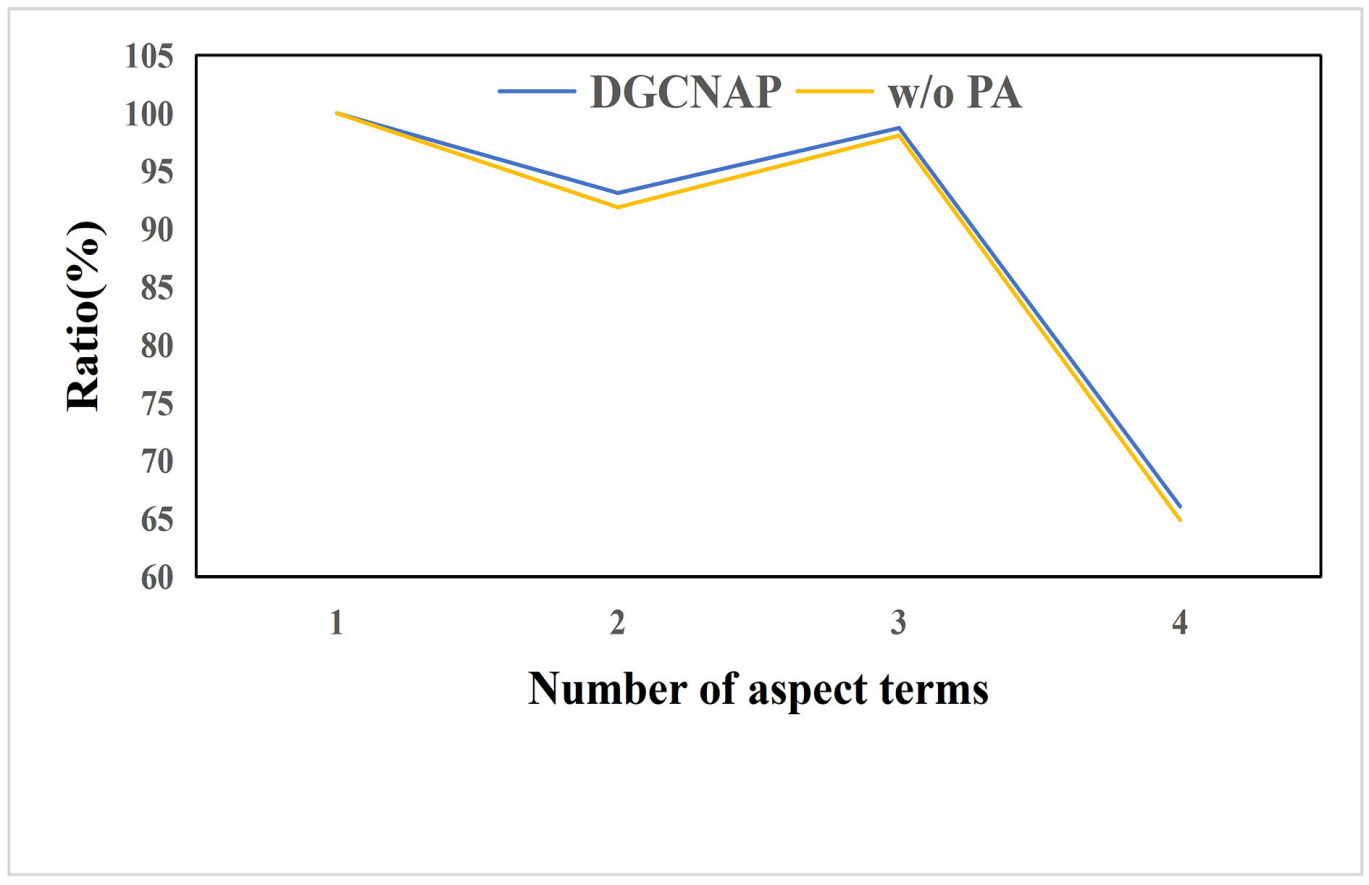
The ratio of F1 value of sentences with multiple aspect words to F1 value of sentences with one aspect word.

To evaluate the effectiveness of the multi-target position-aware function in sentences with multiple triplets, we conduct experiments on sentences with varying numbers of aspect terms on ASTE-data-V2 datasets using Bi-LSTM encoder. Since the number of sentences with multiple aspect terms in the lap14, res15, and res16 datasets is limited, we conduct experiments on the res14 dataset of ASTE-data-V2 using Bi-LSTM encoder. The experimental results are presented in Table 8, and the ratios of the F1 score value of sentences with multiple aspect terms to the F1 score value of sentences with one aspect term are plotted in Figure 6. The results indicate that the implementation of the multi-target position-aware function has a positive impact on the model's ability to handle sentences with multiple triplets. Specifically, as the number of aspect terms increases, the decline rate of the F1 score value is observed to decrease slower than before implementing the function.

**Table 8:** Results of the impact of position-aware function study under the metric of F1.

**Model**	**Number of aspect terms**			
	**1**	**2**	**3**	**4**
DGCNAP	66.26	61.70	65.42	43.77
w/o PA	64.44	59.21	63.20	41.81

### 4.9. Case study

To show the advantages and disadvantages of DGCNAP, a case study is conducted to compare its performance with that of the GTS model. The results of the study are presented in Table 9. The first sample of the study comprises two triplets, with identical opinion terms. GTS accurately predict only one triplet, while DGCNAP successfully identifies both triplets. The second sample also contains two triplets, but GTS make an erroneous identification of a verb as an opinion term, leading to the prediction of an additional triplet based on the incorrect opinion term. In contrast, DGCNAP accurately recognizes the number of aspect terms and make correct predictions for all triplets. The third sample comprises one triplet. However, due to the fact that GTS does not consider contextual affective knowledge information, it inaccurately determine the sentiment polarity of this triplet. In contrast, DGCNAP accurately predict the sentiment polarity by utilizing the affective knowledge information of each word.

**Table 9:** Results of case study.

**Example**	**Golden truth**	**GTS**	**DGCNAP**
Once we sailed, the top-notch food and live entertainment sold us on a unforgettable evening.	(Food, top-notch, positive)(Live entertainment, top-notch, positive)	(Food, top-notch, positive)	(food, top-notch, positive) (live entertainment, top-notch, positive)
If you're craving some serious Indian food and desire a cozy ambiance, this is quiet and exquisite choice.	(Ambiance, cozy, positive)(Indian food, serious, positive)	(Ambiance, cozy, positive)(Indian food, serious, positive)(Indian food, craving, positive)	(Ambiance, cozy, positive)(Indian food, serious, positive)
One caveat: Some of the curried casseroles can be a trifle harsh.	(Curried casseroles, neural)	(Curried casseroles, positive)	(Curried casseroles, neural)

## 5. Conclusion

This study proposes a novel Dual Graph Convolutional Networks Integrating Affective Knowledge and Position Information (DGCNAP) to the ASTE task, which leverages the contextual features, the affective knowledge information of a single word, and relationship between potential multiple triplets in a same sentence. Specifically, our approach utilize two parallel channels to learn relevant features of potential aspect words and potential opinion words, respectively, by incorporating the SenticNet effective score and the weight of potential aspect words or opinion words when constructing the adjacency matrix. Furthermore, a novel multi-target position-aware function is utilized in the GCN Layer to significantly improve the effectiveness of the model in processing sentences with multiple triplets. The experimental results on four benchmark datasets show the effectiveness of DGCNAP, as it outperforms all other state-of-the-art models significantly in terms of F1 on all datasets. Our analysis on the impact of SenticNet Effective Score and Position-aware Function has demonstrated that these improvements effectively increase the model's ability to identify triplets in sentences. Furthermore, supporting the introduction of affective knowledge can enhance the model's ability to recognize sentiment polarity, while introducing a novel multi-target position-aware function can enhance the interaction between triplets and avoid the impact of noise.

It is noteworthy that one aspect may be associated with multiple opinions and vice versa, and our study has not made improvements to address such situations. For future studies, recognition approaches for handling overlapping triplets will be considered. Additionally, an interactive module will be developed to effectively combine enhancement features of both aspect terms and opinion terms.

## Data availability statement

The original contributions presented in the study are included in the article/supplementary material, further inquiries can be directed to the corresponding author.

## Author contributions

YL: conceptualization. YL and QH: methodology and writing. QH and DZ: funding acquisition and supervision. All authors contributed to manuscript revision, read, and approved the submitted version.
